# Recovery of Aromatics from Orange Juice Evaporator Condensate Streams by Reverse Osmosis

**DOI:** 10.3390/membranes10050092

**Published:** 2020-05-08

**Authors:** Fitim Destani, Attilio Naccarato, Antonio Tagarelli, Alfredo Cassano

**Affiliations:** 1 Institute on Membrane Technology, ITM-CNR, c/o University of Calabria, via Pietro Bucci, 17/C-87036 Rende (CS), Italy; f.destani@itm.cnr.it; 2 Department of Chemistry and Chemical Technologies, c/o University of Calabria, via Pietro Bucci, 12/C-87036 Rende (CS), Italy; attilio.naccarato@iia.cnr.it (A.N.); antonio.tagarelli@unical.it (A.T.)

**Keywords:** evaporator condensates, citrus processing, aroma compounds, reverse osmosis (RO)

## Abstract

The aim of this work was to analyze the potential of reverse osmosis (RO) membranes in the recovery and concentration of aroma compounds from orange juice evaporator condensate (EC) streams. Concentration experiments were performed by using three RO spiral-wound aromatic polyamide membranes (SG1812C-34D, SC1812C-34D and SE1812) with different NaCl rejections. The effect of transmembrane pressure, axial feed flowrate and volume concentration ratio (VCR) on permeate flux was studied. Rejections of the investigated membranes towards specific aroma compounds (octanol, α-terpineol, terpinen-4-ol, cis-carveol, karvon, linalool) in selected operating conditions were also evaluated. The concentrations of the aroma compounds were determined by gas chromatography coupled with mass spectrometry (GC-MS) using headspace solid-phase microextraction (HS-SPME) as a sample preparation approach. For all selected membranes, the permeate flux increased linearly by increasing the operating pressure from 5 to 25 bar; on the other hand, the feed flowrate did not have any significant effect on the permeate flux. High retention values towards aroma compounds (>80%) were measured for all selected membranes. However, the SC membrane showed the highest rejection values (>96%) and the best correlation between concentration factor of aroma compounds and VCR.

## 1. Introduction

The citrus processing industry is characterized by the production of a considerable amount of by-products of commercial value including dried pulp and molasses, juice pulps and pulp wash (secondary juice) and d-limonene [1]. These by-products represent a serious environmental problem, since the plant material is usually prone to microbial spoilage and is commonly used in animal feed or as fertilizers. However, most of these materials are enriched in bioactive compounds, such as flavonoids and phenolic acids, recognized for their beneficial implications in human health due to their antioxidant activity and free radical scavenging ability [2]. Therefore, they have a great interest in food, pharmaceutical and cosmeceutical applications, because of their valuable technological and nutritional properties [3].

In this context, evaporator condensate (EC) streams, produced in the juice concentration step by multiple-effect evaporation, represent the largest volume generated in the process. They are used by the industries in many ways, including fruit cleaning/washing, extraction/recovery of solids residual of the pulp, water replacement in the process of peel essential oil recovery, calories recovery for use in boilers, floor, equipment and restrooms cleaning [4]. It has been estimated that each kg of frozen juice produces, theoretically, about 4.9 kg of EC streams.

The composition of this waste stream is contingent on the efficiency of the concentration process. Considering that the efficiency of the concentration process is less than 100% (all non-water constituents are desirably retained with the concentrated juice), it results in a waste stream mainly constituted by water carrying over constituents, such as aroma and other volatiles, organic acids and sugars. Among them, aroma compounds have typical odors and have a particularly low detection threshold (about a few micrograms per cubic meter). These compounds are widely used as additives for flavoring foods and medicines or in the perfume and cosmetic industries. In addition, they have been shown to possess antimicrobial [5] and antioxidant activity [6]. Therefore, EC streams may constitute, with selective treatment, a source of molecules of great industrial interest [7,8].

The potential reuse of EC streams in other industrial applications has been largely under-investigated in the existing literature. Coelho et al. [9] reported that the use of ultrafiltration with cellulose acetate membranes of 30 kDa at 1 bar was effective to remove color and turbidity of recovered water of concentrate orange juice, within the maximum limits established by the Brazilian legislation for bottled water.

According to the method patented by Destefano [10], aroma compounds can be recovered from aqueous condensates by high temperature distillation; however, this technology is an energy-intensive, expensive process, especially with these streams that contain relatively small concentrations of organic constituents in large volumes of water. Another specific problem, particularly when handling citrus essences, is that the high temperatures involved in distillation may destroy the oily portion of the essence, with a resulting loss of quality.

Membrane processes, including pervaporation (PV) [11,12,13,14,15], vacuum membrane distillation (VCMD) [16,17,18] and reverse osmosis (RO) [19,20,21] represent a valid alternative to the use of thermal distillation, since they use gentle temperatures during the operation and do not involve phase change or chemical additives. In addition, they are characterized by low energy consumption and easy scale-up [22]. In particular, RO is an effective technology to remove organic compounds with low molecular weight and low concentration from aqueous solutions in different fields, including fruit and vegetable juice processing [23]. This technology can effectively double the operating capacity and improve both color and flavor characteristics when compared with thermal evaporation and/or freeze concentration [24].

The retention of juice constituents, especially flavors, and the permeate flux, regarding RO performance, are two major factors, which are related to the type of membranes and the operating conditions used during the process. Early studies by Merson and Morgan [25] found that oil-soluble aromas of orange juice were easily retained by using cellulose acetate (CA) membrane, while there were losses of water-soluble aromas. The production of new polymer membranes, such as those in polyamide (PA), allowed a rapid development of the RO application also in this field. Indeed, PA membranes exhibited higher fluxes and flavor retention when compared to CA membranes [26]. Apple flavor retentions of more than 80% were measured when using high resistance polyamide membranes supported on polysulphone membranes [27]. The preferential sorption-capillary flux model was proposed by Álvarez et al. [20], to predict the rejection of aroma compounds and permeate flux in the concentration of a model solution simulating apple juice through a spiral-wound aromatic polyamide membrane. Authors observed that permeate flux and aroma rejection increased with feed flowrate and decreased with sugar concentration. Recently, Tremblay et al. [28] designed and optimized a membrane process based on the use of a RO spiral-wound polyamide membrane to concentrate flavor compounds from cooking effluents generated by snow crab processing facilities, usually considered as waste. The membrane retained almost dissolved solids in the effluent (mainly proteins and minerals), including flavor compounds.

To the best of our knowledge, there is no report in the scientific literature concerning the application of membrane processes for the recovery of aroma compounds from orange juice EC streams. Therefore, this work was aimed at evaluating the performance of RO membranes for the selective removal and concentration of aroma compounds from EC streams, in order to produce aromatic fractions of interest in food, cosmeceutical and pharmaceutical applications. Another interesting perspective of the experimental study is the possibility to produce fresh water as RO permeate, thus allowing one to save water resources. Experimental runs were performed on a laboratory scale by using three different polyamide membranes in spiral-wound configuration (SG1812C-34D, SC1812C-34D and SE1812, all from GE Water & Process Technologies), with NaCl rejections in the range 98%–99.5%. The effect of transmembrane pressure, axial feed flowrate and volume concentration ratio on permeate flux was investigated. Rejections of aroma compounds (octanol, α-terpineol, terpinen-4-ol, cis-carveol, karvon, linalool) were evaluated in batch concentration experiments performed in selected operating conditions.

## 2. Materials and Methods

### 2.1. Solutions and Reactants

Orange juice EC streams were provided by Gioia Succhi Srl (Rosarno, RC, Italy). Before the RO treatment, condensed streams were filtered through a 200 µm cotton fabric filter in order to remove foreign materials.

Moreover, 2,6-dimethyl-2,4,6-octatriene standard and 85 μm carboxen/polydimethylsiloxane SPME fiber were purchased from Sigma-Aldrich (Milan, Italy).

### 2.2. Reverse Osmosis Equipment and Procedures

**Figure 1:**
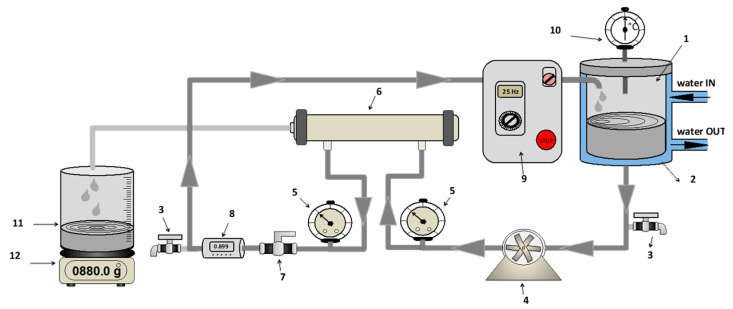
Schematic representation of reverse osmosis (RO) plant (1—feed tank; 2—cooling shell; 3—discharge tap; 4—feed pump; 5—manometers; 6—membrane module; 7—regulation valve; 8—digital flowmeter; 9—control panel; 10—thermometer; 11—permeate tank; 12—digital balance).

Experimental runs were performed by using a RO laboratory bench plant consisting of a control panel, a cylindrical jacketed feed tank (with a capacity of 5L) constructed from stainless steel (SS 316), a feed plunger pump with belt drive (Cat Pumps, Milano-Italy, Model 3CP1221), two pressure gauges (Wika, Lawrenceville, GA, USA) (max pressure 100 bar, absolute error 1 bar), a digital flow meter (SM6000, IFM Electronic GmbH, Hamburg, Germany), a thermometer placed inside the feed tank and a cylindrical housing able to accommodate a 11.74 × 1.75 inches spiral wound membrane module (Figure 1). The adjustment of operating pressure and feed flowrate was done by simultaneously pump rotation control through a frequency inverter and a needle valve. The operating temperature was controlled by circulating either a heating fluid or a coolant through the tank jacket.

Three different spiral-wound membranes (SG1812C-34D, SE1812C-34D and SC1812), all supplied by GE Water & Process Technologies (Minnetonka, MN, USA), were tested. All membranes were in polyamide with different NaCl rejections (98%, 99% and 99.5%, respectively).

RO experiments were performed according to both total recycle and batch concentration configuration. In the first option, both permeate and retentate streams were recycled back to the feed reservoir with a constant feed volume. This configuration was used to evaluate the effect of the transmembrane pressure (TMP) and feed flowrate (*Q_f_*) on the permeate flux. Experiments were performed at an operating temperature of 20 ± 1 °C. TMP values were modified in the range 5–25 bar at a *Q_f_* of 360 L/h; the effect of *Q_f_* on the permeate flux was investigated in the range 75–825 L/h at fixed values of TMP (5 bar). All tests were conducted for 60 min.

Batch concentration experiments were performed by separately collecting the permeate stream and recycling back the retentate in the feed tank in selected operating conditions (TMP, 25 bar; *Q_f_*, 360 L/h; temperature, 20 ± 1 °C), up to a volume concentration ratio (VCR) of 7.5.

VCR is defined according to Equation (1):

(1) $$VCR=\frac{V_{f}}{V_{r}}=1+\frac{V_{p}}{V_{r}}$$

where *V_f_*, *V_r_* and *V_p_* are the volume of the feed, retentate and permeate solutions, respectively.

The volumetric flux of permeate, *J_p_* (L/m^2^h), was calculated by measuring the permeate volume collected in a certain time, according to the following equation:

(2) $$J_{p}=\frac{V_{p}}{A\cdot t}$$

where *V_p_* (L) is the permeate volume at time *t* (h) and *A* is the membrane effective area (m^2^).

The water permeability of each membrane was obtained as the slope of the straight line resulting from plotting the water flux versus the applied TMP (at 20 ± 1 °C and a *Q_f_* of 360 L/h).

### 2.3. Analytical Method

Feed, permeate and retentate samples coming from RO experiments were collected and stored at −20 °C for further analyses.

The concentrations of the aroma compounds were analyzed in the permeate, retentate, and feed streams by gas chromatography coupled with mass spectrometry (GC-MS), using headspace solid-phase microextraction (HS-SPME) as a convenient and suitable sample preparation approach, which is widely used in several application fields [29,30,31,32]. SPME was performed using an 85 μm carboxen/polydimethylsiloxane fiber, which demonstrated to be a suitable coating for the assay of small volatiles [33]. For the analysis, 2 mL of sample were transferred into a vial and then fortified at 10 µg/L with 2,6-dimethyl-2,4,6-octatriene (alloocimene), which was used as an internal standard for quantitative analysis. Each vial was crimped with a septum and HS-SPME was carried out by exposing the fiber in the headspace volume for 30 min at room temperature. The adsorbed analytes were thermally desorbed by introducing the fiber into the GC injector, set at 250 °C for 5 min. Thermal blanks of the fiber were periodically carried out to verify the potential presence of memory effect.

The analyses were carried out in full scan mode using a TSQ Quantum GC (Thermo Fischer Scientific, Waltham, USA) system, constituted by a triple quadrupole mass spectrometer (QqQ) Quantum and a TRACE GC Ultra equipped with a TriPlus autosampler for fully automated SPME extraction.

Chromatographic separation of the analytes was performed using a Restek Rxi-5MS capillary column (30 m × 0.25 mm i.d., 0.25 µm film thickness, 95% polydimethylsiloxane, 5% polydiphenylsiloxane).

The temperature of the GC oven was initially held at 40 °C for 3 min, then ramped at 1 °C/min to 80 °C, ramped again at 20 °C/min to 250 °C, and held for 2.5 min. The carrier gas was helium flowing at 1 mL/min and the GC injector performed in splitless mode using a Thermo PTV straight Liner 0.75 × 2.75 × 105 mm. The volatile molecules were identified by comparing the mass spectra with reference spectra from the NIST 02 database (NIST/EPA/NIH Mass Spectral Library, version 2.0, Gaithersburg, MD, USA).

Properties of identified aroma compounds and their concentrations in the evaporated water of thermally evaporated orange juice are reported in Table 1.

All measurements were made in triplicate. Results were expressed as mean value ± SD.

The quantitative assay of each compound was performed using the peak area given by the characteristic ionic species, i.e., octanol m/z 56; α-terpineol m/z 59; terpinen-4-ol m/z 71 and 111; cis-carveol m/z 84; karvon m/z 82; linalool m/z 71 and 121; 2,6-dimethyl-2,4,6-octatriene m/z 121.

The rejection (*R*) of RO membranes towards specific aroma compounds was calculated as follows:

(3) $$R\left(\%\right)=\left(1-\frac{C_{p}}{C_{f}}\right)\cdot 100$$

where *C_p_* and *C_f_* are the solute concentration in permeate and feed, respectively.

## 3. Results and Discussion

### 3.1. Effect of TMP and Feed Flowrate on Permeate Flux

**Figure 2:**
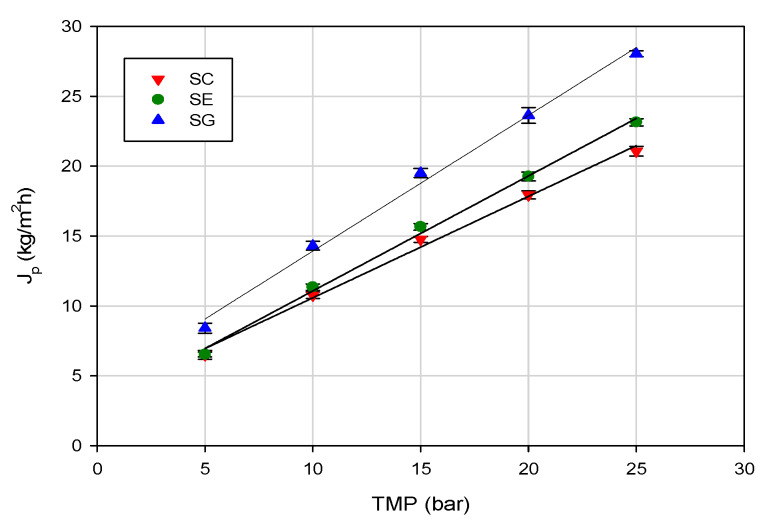
RO of evaporator condensate stream. Effect of transmembrane pressure (TMP) on steady-state permeate flux (operating conditions: temperature, 20 ± 1 °C; feed flowrate, 360 L/h).

The influence of TMP on the permeate flux for the investigated membranes in selected operating conditions is shown in Figure 2. Experimental data indicated a linear dependence of pressure influence on the permeate flux due to minimal concentration polarization and fouling phenomena. Similar results were obtained in the treatment of ethanol, aldehyde and ester solutions at different concentration (in the range 0.01%–0.1%) by using composite membranes (high-resistance polyamide supported on polysulphone membranes) [34].

**Figure 3:**
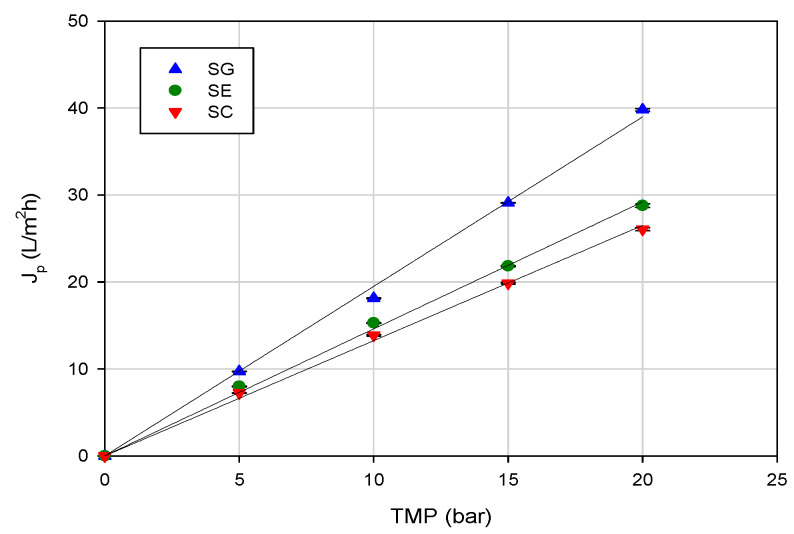
Water flux versus transmembrane pressure for selected RO membranes (operating conditions: temperature, 20 ± 1 °C; feed flowrate, 360 L/h).

In the range of investigated operating pressures, the SG membrane, with the lowest NaCl rejection, exhibited higher permeation fluxes when compared with the other two investigated membranes. This behavior was in agreement with the measurement of pure water flux. Indeed, at an operating temperature of 20 °C, the SG membrane showed the highest water permeability (1.94 L/m^2^hbar), in comparison with the SE (1.46 L/m^2^hbar) and SC (1.32 L/m^2^hbar) membranes (Figure 3).

**Figure 4:**
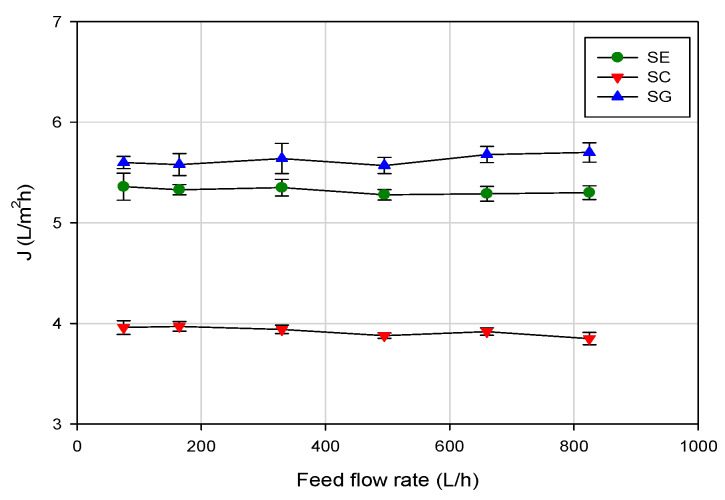
RO of evaporator condensate stream. Effect of feed flowrate on steady-state permeate flux (operating conditions: temperature, 20 ± 1 °C; TMP, 5 bar).

Figure 4 shows the effect of the feed flowrate on the permeate flux at an operating pressure of 5 bar and at 20 ± 1 °C. For all the selected membranes, an increase in the feed flowrate in the range of investigated values (100–800 L/h) did not produce any significant increase of the permeate flux. This result can be explained assuming that the water flux was not strongly affected by the hydrodynamic conditions of the feed solution, containing a very low concentration of aroma compounds. In these conditions the effect of concentration polarization on permeate flux can be considered negligible.

Adversely, a little increase of the permeate flux with feed flowrate was observed by Alvarez et al. [20] in the RO concentration of apple juice model solutions containing a higher concentration of aroma compounds (of the order of 10–200 ppm for each selected aroma compound), with a spiral-wound aromatic polyamide membrane (MSCB 2521 R99, from Separem Spa, Biella, Italy). This behavior was attributed to the reduction of concentration polarization with feed flowrate, because of better mixing in the high-pressure channel.

### 3.2. Effect of VCR on Permeate Flux

**Figure 5:**
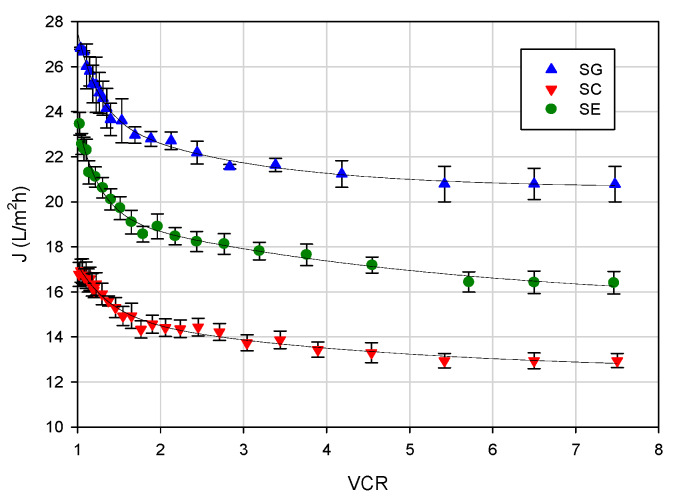
RO of evaporator condensate stream. Effect of volume concentration ratio (VCR) on the permeate flux (operating conditions: temperature, 20 ± 1 °C; feed flowrate, 360 L/h; TMP, 25 bar).

Figure 5 shows the effect of the VCR on the permeate flux at an operating temperature of 20 ± 1 °C, an axial feed flowrate of 360 L/h and a TMP of 25 bar. The flux vs. VCR curve can be divided into three steps: a first step up to VCR 2 characterized by a rapid decrease of the permeate flux; a second step up to VCR 4 characterized by a slow decline of the permeate flux; a third step characterized by a steady-state permeate flux value. In particular, permeate fluxes decreased of about 22% for SG and SE membranes and 30% for the SC membrane, although during the set-up of the transmembrane pressure, the permeate fluxes increased linearly with pressure for all selected membranes. This behavior can be attributed to the increased concentration of the EC stream in the feed reservoir according to the batch concentration configuration. As the feed concentration increases, the concentration polarization becomes more severe. More solutes are convected towards the membrane surface, resulting in a thicker cake layer, which increases the resistance against the solvent flux [35]. In addition, the increased concentration of solutes increases the osmotic pressure, which at constant TMP causes the flux through the membrane to decrease. These results are in agreement with those reported by Tremblay et al. [28] in the concentration of snow crab cooking effluents with a polyamide RO membrane in spiral-wound configuration, having a 99% NaCl rejection (AG1812C-34D from GE Power, Gloucester, ON, Canada).

In agreement with the data of salt rejection, the SG membrane (NaCl rejection of 98%) exhibited the highest productivity, with a steady-state flux of about 20.8 kg/m^2^h; for the SE and SC membranes (NaCl rejections of 99% and 99.5%, respectively), the steady-state fluxes were of 16.4 and 12.9 kg/m^2^h, respectively.

### 3.3. Retention of Aroma Compounds

**Figure 6:**
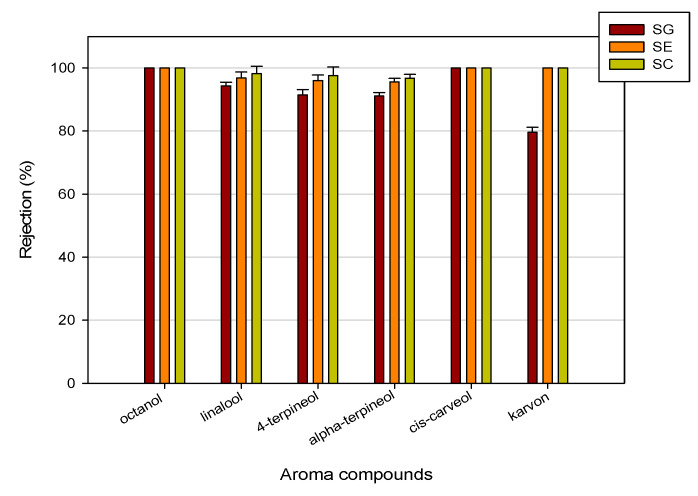
Rejection of RO membranes towards aroma compounds.

In Table 2, the analyses of aromatic compounds in feed and permeate streams of condensed water from thermally evaporated orange juice treated with RO membranes are reported. Data are referred to experimental runs performed in batch concentration at 25 bar, 20 °C and an axial feed flowrate of 360 L/h, up to a VCR of 7.5. For all selected membranes, octanol and cis-carveol were not detected in the permeate stream. For other aromatic compounds, the SG membrane showed a lower retention in comparison to the SE and SC membranes, according to its lower NaCl rejection (98%). In particular, for both SE and SC membranes, the rejection towards aroma compounds was higher than 95% (Figure 6). In general, the retention of aroma compounds can be reasonably well explained by the polar, steric and nonpolar effects of the solute molecules. In particular, it is well known that the recovery of aroma compounds in the retentate of RO membranes is strongly affected by the molecular mass [36]. In our case, the observed rejection with RO membranes are of the same order of magnitude, considering that the molecular mass and the polarity of the analysed aromas are very similar.

High retention values (>80%) were also observed by Alvarez et al. [20] and Schutte [37] for alcoholic aroma compounds (butanol, hexanol, isopentanol, isobutanol) in the concentration of model solutions with aromatic polyamide RO membranes.

**Figure 7:**
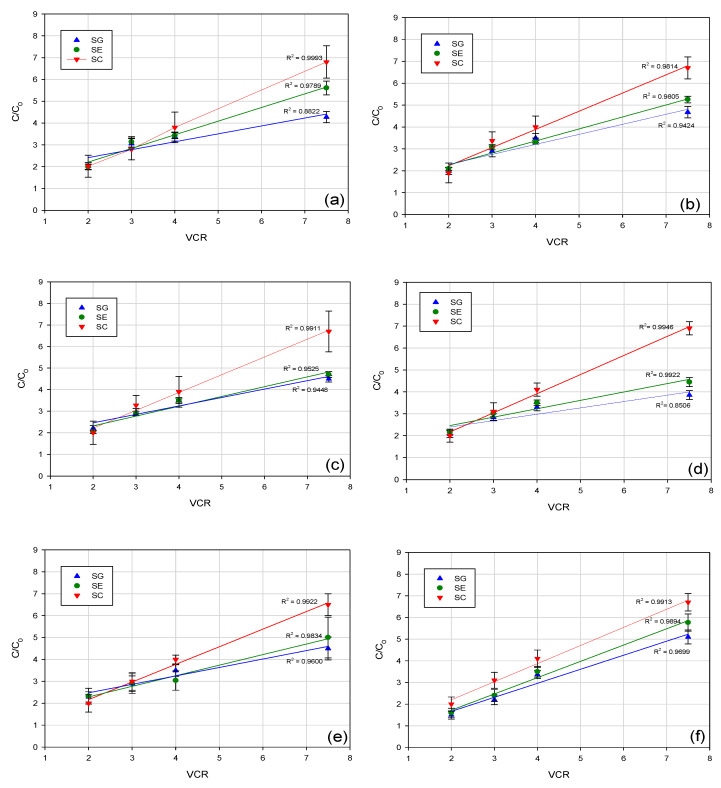
Profile of concentration factor with volume concentration ratio (VCR) for: (**a**) octanol; (**b**) linalool; (**c**) terpinen-4-ol; (**d**) α-terpineol; (**e**) cis-carveol; (**f**) karvon.

The profile of concentration factor for all investigated aroma compounds as a function of VCR is reported in Figure 7. Experimental results indicated for the SC membrane the best correlation coefficient (more than 0.99), between concentration factor of aroma compounds and VCR; in particular, for all investigated compounds an increase of their concentration up to 6.7–6.9 fold in comparison with the feed solution was detected at a VCR of 7.5. For SG and SE membranes, the concentration of aroma compounds at VCR 7.5 resulted in being 4 to 5.5-fold higher than the feed solution, indicating a partial loss of these compounds during the process. This phenomenon can be partially attributed to their permeation through the membrane (SG and SE membranes exhibited a lower retention towards most of the analyzed compounds in comparison to the SC membrane), although interactions with the membrane material (membrane adsorption) cannot be excluded. In addition, for all selected membranes, loss of aroma compounds from the retentate due to volatilization appeared to be significant, starting from a VCR 4, due to the increased time of processing.

These results are in agreement with those observed by Pozderović et al. [36] in the concentration of alcohol model solutions with RO composite membranes (HR 95 PP and HR 98 PP from Dow Denmark Separation Systems, Copenhagen, Denmark). Authors observed that the decreasing processing time resulted in a lower loss of aroma compounds from the retentate, due to volatilization and membrane adsorption.

The whole results indicate that the SC membrane is the most suitable membrane to recover and concentrate aroma compounds from EC streams produced in the concentration of orange juice. The concentrated solution could be of interest for food applications (i.e., aroma compounds for food and beverages), as well as for the formulation of functional foods or cosmeceutical products. On the other hand, the purified water stream (permeate) can be reused in the orange juice production as process water (cleaning of fruits and plants, cleaning of membranes, irrigation, etc.), with additional advantages in terms of water saving and the reduction of environmental pollution.

## 4. Conclusions

The recovery and concentration of aroma compounds from evaporated water produced in the thermal concentration of orange juice was investigated through the use of reverse osmosis membranes in spiral-wound configurations with different NaCl rejections. For all selected membranes, the permeate flux was observed to increase with the transmembrane pressure, in the range of investigated operating values (5–25 bar). On the other hand, an increase in the feed flowrate in the range of 100–800 L/h did not produce any significant increase of the permeate flux.

In agreement with the data of salt rejection, the SG membrane (NaCl rejection 98%) exhibited the highest productivity (steady-state flux of about 20.8 kg/m^2^h) in batch concentration experiments performed in selected operating conditions, up to a volume concentration ratio of 7.5. However, by referring to the membrane selectivity, the SC membrane (NaCl rejection 99.5%) showed the highest retention of aroma compounds (higher than 96%) with the total rejection of octanol, cis-carveol and karvon. This membrane also showed the best correlation between the concentration factor of aroma compounds and VCR.

The results of this study would lay the experimental basis for a potential exploitation of the orange juice evaporator condensate streams, through the production of concentrated aroma solutions of interest in food applications or as functional ingredients (i.e., drinks of nutritional water enriched with orange aroma). They also provide a useful approach for producing fresh water to be reused for different purposes, thus saving water resources.

## Figures and Tables

**Table 1:** Properties of aroma compounds and their concentration in condensed water from thermally evaporated orange juice.

Compound	Concentration ^a^ (µg/L)	Molecular Weight (g/mol)	Molecular Formula	Structure	Density (g/cm^3^)	Water Solubility at 25 °C (g/L)
octanol	18.3–35.8	130.23	C_8_H_18_O	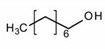	0.8262	0.54
α-terpineol	47.0–77.1	154.25	C_10_H_18_O	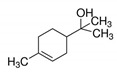	0.935	2.42
terpinen-4-ol	50.4–97.0	154.25	C_10_H_18_O	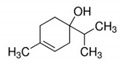	0.926	2.5
cis-carveol	2.31–3.83	152.23	C_12_H_18_O_2_	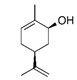	0.958	2.82
karvon	5.41–9.83	150.21	C_10_H_14_O	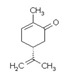	-	1.30
linalool	44.7–86.5	154.25	C_10_H_18_O	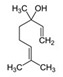	0.858	1.589

^a^ Minimum and maximum values detected in three feed samples treated with the selected membranes.

**Table 2:** Analyses of aromatic compounds in feed and permeate streams of condensed water from thermally evaporated orange juice treated by RO membranes.

Compound	Membrane Type							
	SG			SE			SC	
	Feed	Permeate		Feed	Permeate		Feed	Permeate
	(μg/L)	(μg/L)		(μg/L)	(μg/L)		(μg/L)	(μg/L)
octanol	26 ± 2	n.d.		18 ± 2	n.d.		36 ± 2	n.d.
linalool	72 ± 6	4.1 ± 0.4		45 ± 5	1.4 ± 0.3		86 ± 3	1.6 ± 0.3
terpinen-4-ol	84 ± 4	7.2 ± 0.6		50 ± 4	2.1 ± 0.3		97 ± 3	2.4 ± 0.2
α-terpineol	77 ± 5	6.9 ± 0.5		47 ± 6	2.1 ± 0.3		84 ± 5	2.7 ± 0.2
cis-carveol	3.5 ± 0.3	n.d.		2.3 ± 0.4	n.d.		3.8 ± 0.6	n.d.
karvon	8.7 ± 0.6	1.8 ± 0.2		5.4 ± 0.2	n.d.		9.8 ± 0.8	n.d.
